# Profiles of *Staphyloccocus aureus* isolated from goat persistent mastitis before and after treatment with enrofloxacin

**DOI:** 10.1186/s12866-020-01793-9

**Published:** 2020-05-24

**Authors:** Magna Coroa Lima, Mariana de Barros, Thalita Moreira Scatamburlo, Richard Costa Polveiro, Laís Karolyne de Castro, Samuel Henrique Sales Guimarães, Sanely Lourenço da Costa, Mateus Matiuzzi da Costa, Maria Aparecida Scatamburlo Moreira

**Affiliations:** 1 Laboratory of Bacterial Diseases (LDBAC), Veterinary Department, Universidade Federal de Vicosa (UFV), PH Rolfs Avenue, s/n, University Campus, Viçosa, Minas Gerais 36570-900 Brazil; 2 Laboratory of Microbiology and animal immunology, Animal Science Department, Universidade Federal do Vale do São Francisco (UNIVASF), José de Sá Maniçoba Street, Center, Petrolina, Pernambuco 56306-410 Brazil

**Keywords:** Caprine mastitis, Small ruminants, Persistence

## Abstract

**Background:**

*Staphylococcus aureus* is one of the main causative agents of mastitis in small ruminants. Antimicrobial use is the major treatment, but there are many flaws linked to resistance, tolerance or persistence. This study aimed to verify changes in resistance, virulence and clonal profiles of *S. aureus* isolated from persistent mastitis goat milk before and after enrofloxacin treatment.

**Results:**

MIC increased to at least one antimicrobial in *S. aureus* isolates after enrofloxacin treatment compared to before. The most detected resistance genes before and after treatment were *tetK*, *tetM*, and *blaZ*, with more resistance genes detected after enrofloxacin treatment (*p* < 0.05). Occasional variations in efflux system gene detection were observed before and after treatment. Nine virulence genes (*hla*, *fnbA*, *fnbB*, *eta*, *etb*, *sea*, *sec*, *seh*, and *sej*) were detected at both times, and between these, the *hla* and *eta* genes were detected more in isolates after treatment. All isolates of *S. aureus* belonged to the same sequence type (ST) 133, except for two *S. aureus* isolates prior to enrofloxacin treatment which were classified as ST5 and the other as a new one, ST4966. Isolates of *S. aureus* 4, 8, and 100 from before and after treatment had identical pulse types, while others obtained from other animals before and after treatment were classified into distinct pulse types.

**Conclusion:**

There were occasional changes in the studied profiles of *S. aureus* isolated before and after treatment of animals with enrofloxacin, which may have contributed to the permanence of bacteria in the mammary gland, even when using traditional treatment, resulting in persistent mastitis.

## Background

Goat farms suffer severe economic losses due to staphylococcal intramammary infections, with *Staphylococcus aureus* being the main cause of clinical mastitis in small ruminants [1]. Intramammary infections caused by *S. aureus* deserve special attention, due to the high prevalence and diverse forms of presentation of the disease. *S. aureus* is responsible for both acute clinical mastitis (gangrenous mastitis) and subclinical mastitis [2].

Mastitis is traditionally treated with the use of antimicrobials; however, the success of this therapy is low in many cases. The use of enrofloxacin in goats and other small ruminants in mastitis treatments has been widely accepted by the main routes of administration and has proved its efficacy in the resolution of mastitis [3, 4]. The phenomena of resistance, tolerance and persistence have brought greater complexity to the flaws of antimicrobial therapies [5]. *S. aureus* possesses different virulence factors that contribute to its persistence in mammary tissue [6]. Besides, the pattern of virulence genes can be used to determine the biovar and the relationship with the origin of the isolates [7]. In addition to virulence, a major concern in the control of mastitis is resistance to antimicrobials of the etiological agent. Finally, the characterization of the genetic diversity of *S. aureus* is important to understand the pattern of dispersion of the pathogen [6].

In this way, the present study aims to verify changes in the clonal, resistance and virulence profiles of *S. aureus* isolated from the milk of goats with persistent mastitis, before and after treatment with enrofloxacin.

## Results

### Resistance profile

The minimum inhibitory concentration (MIC) values are shown in Table 1. Considering the same animal, a MIC increase to at least one antimicrobial was observed for all *S. aureus* isolates after treatment, compared with the values found before it. This was observed in a greater number of isolates for enrofloxacin, ciprofloxacin, and oxacillin MIC values, followed by penicillin, gentamicin, and ampicillin, then by vancomycin and lastly by tetracycline MICs (Table 1, in bold).

**Table 1:** Values of Minimum Inhibitory Concentration of different antimicrobials in *Staphylococcus aureus* isolates using the E-test®

Isolates	GEN	TET	VAN	ENR	CIP	OXA	PEN	AMP
	*(R ≥ 16)*	*(R ≥ 16)*	*(R ≥ 16)*	*(R ≥ 8)*	*(R ≥ 4)*	*(R ≥ 4)*	*(R ≥ 0, 25)*	(R ≥ 2*)
4^c^	3	125	0,25	0,19	3	24	24	3
4^p^	**6**	32	0,145	**0,75**	3	24	24	**16**
5^c^	12	64	3	0,38	3	24	16	12
5^p^	12	16	3	**1,25**	**12**^**1**^	**32**	16	12
6^c^	3	16	1,5	0,38	96	32	8	16
6^p^	**6**	**24**	**16**^**1**^	**0,75**	96	**42**	**96**	**24**
7^c^	4	36	18	0,125	0,25	24	24	12
7^p^	**8**	32	18	**0,19**	**0,48**	24	24	12
8^c^	3	96	6	0,25	0,64	12	12	2
8^p^	**4**	96	**12**	0,25	**3**	**32**	**32**	2
9^c^	1	96	32	0,19	3	32	32	4
9^p^	0,5	96	32	**0,5**	3	**48**	**38**	4
10^c^	12	48	48	0,75	3	48	48	48
10^p^	3	48	48	0,75	**4**^**1**^	**96**	**96**	48
100^c^	4	48	48	0,5	2	12	12	12
100^p^	2	48	**96**	0,5	**12**^**1**^	12	12	**16**
101^c^	48	18	3	16	6	96	0,75	16
101^p^	48	18	3	16	6	96	0,75	**18**

Values of MIC in micrograms per milliliter (μg/mL) of different antimicrobials in *Staphylococcus aureus* isolated from goats with mastitis before (c) and after (p) enrofloxacin treatment using the E-test® (bioMerieux). *R* resistance. *GEN* Gentamicin; *TET* Tetracycline; *VAN* Vancomycin; *ENR* Enrofloxacin; *CIP* Ciprofloxacin; *OXA* Oxacillin; *PEN* Penicillin, *AMP* Ampicillin. Underlined number: resistant for the antimicrobial in test. Bold number: MIC value increased in the isolates obtained in the same animal prior and after enrofloxacin treatment. ^1^Sensitivity profile change for resistance in the isolates obtained in the same animal prior and after enrofloxacin treatment. Breakpoint: CLSI [8]. *Breakpoint MacGowan and Wise [9]

According to the cutoff points of the Clinical and Laboratory Standards Institute [8] and Macgowan and Wise [9], all isolates showed resistance profiles for tetracycline, penicillin, ampicillin and oxacycline, while for the other antimicrobials there were variations (Table 1). In addition, after treatment some *S. aureus* isolates changed the profile for antimicrobial resistant to vancomycin and ciprofloxacin, as highlighted in Table 1.

**Table 2:** Virulence and resistance genes detected in *Staphylococcus aureus* isolates from mastitis goat milk

Isolates	Resistance profile	Virulence profile
4 ^c^	*blaZ*, *ermA*, *mecA*, *tetK*, *tetM*, ***lmrS***, ***norA***, ***norC***, ***tet38***	*fnbA, fnbB, hla*
4 ^p^	*blaZ*, *ermA*, *mecA*, *tetK*, *tetM*, ***lmrS***, ***norA***, ***norC***, ***tet38***	*fnbA, fnbB, hla, eta*
5 ^c^	*erma, tetK, tetM,****norA, norC, tet38***	*eta, fnbB, sea, sej*
5 ^p^	*ant(4′)-Ia, tetK, tetM.,****lmrS, norA, norC, tet38***	*eta, fnbB, hla, sea, sej,*
6 ^c^	*ant(4′)-Ia, blaZ, tetM, tetK,****norA, norC, tet38***	*etb, sec, sej*
6 ^p^	*ant(4′)-Ia, blaZ,,tetM, tetK****, norA, norC, tet38***	*etb, sec, sej*
7 ^c^	*blaZ,****norA, norC, tet38***	*fnbA, fnbB, hla*
7 ^p^	*ant(4′)-Ia, blaZ, ermB, tetM, tetK,****lmrS, norA, norC, tet38***	*fnbA, fnbB, hla*
8 ^c^	*ermB,****lmrS, norC, tet38***	*etb, hla*
8 ^p^	*ermB,****lmrS, norC, tet38***	*etb, hla*
9 ^c^	*blaZ, mecA*	*etb, sea,*
9 ^p^	*blaZ, mecA, tetM, tetK,****norA, norC, tet38***	*etb, hla, sea*
10 ^c^	*ant(4′)-Ia****norC, tet38***	*hla, seh*
10 ^p^	*ant(4′)-Ia,****norC, tet38***	*hla, seh*
100 ^c^	*tetK, tetM,****norC, tet38***	*fnbA, hla, sec*
100 ^p^	*tetK, tetM,****norC, tet38***	*etb, fnbA, hla, sec*
101 ^c^	*ant(4′)-Ia, blaZ, tetM, tetK,****norA, norC, tet38***	*etb, fnbB, hla, sec*
101 ^p^	*ant(4′)-Ia, blaZ, tetM, tetK,****norA, norC, tet38***	*etb, fnbB, hla, sec*

c: isolates of goats with mastitis before treatment; p: isolates of goats with mastitis after treatment. Bold: multidrug efflux system genes

The most frequently detected resistance genes in *S aureus* isolates before and after treatment were *tetK, tetM* and *blaZ* (Table 2). More resistance genes were detected in the isolates obtained after treatment with enrofloxacin (60.87%, 28/46) compared to those detected in *S. aureus* isolated prior to treatment (39.13%, 18/46) (*P* < 0.05). The genes *aac(6‘)/aph(2’), aph(3′)-llla* and *ermC* were not detected at both moments.

*S. aureus* from animals 7 and 9 showed greater variation in the amount of resistance genes detected before and after treatment (Table 2).

Regarding multidrug efflux systems genes, *norC* and *tet38* were the most prevalent, being found in 17 of 18 isolates (94.4%) (Table 2). *S. aureus* from animal 9 varied the most: there was no gene before treatment and after treatment it was positive for three genes. Moreover, in isolates from animals 5 and 7, after treatment the *lmrS* gene was detected in addition to the genes that were detected also before treatment. The *norB*, *mgrA* and *msrA* genes were not detected in *S. aureus* isolated at the two studied moments.

### Virulence profile

The detection of *S. aureus* virulence genes isolated from goat milk with clinical mastitis before and after treatment with enrofloxacin are shown in Table 2. Of the 16 genes tested, only nine genes (*hla, fnbA, fnbB, eta, etb, sea, sec, seh and sej*) were detected in *S. aureus* isolated before and after enrofloxacin treatment. Among these, the genes *hla* (alpha-hemolysin) and *eta* (exfoliative toxin A) were more detected in *S. aureus* isolated after treatment. However, there were no statistical difference. The others, related to adhesion and toxins, remained constant at both times.

### Clonal profile

**Fig. 1:**
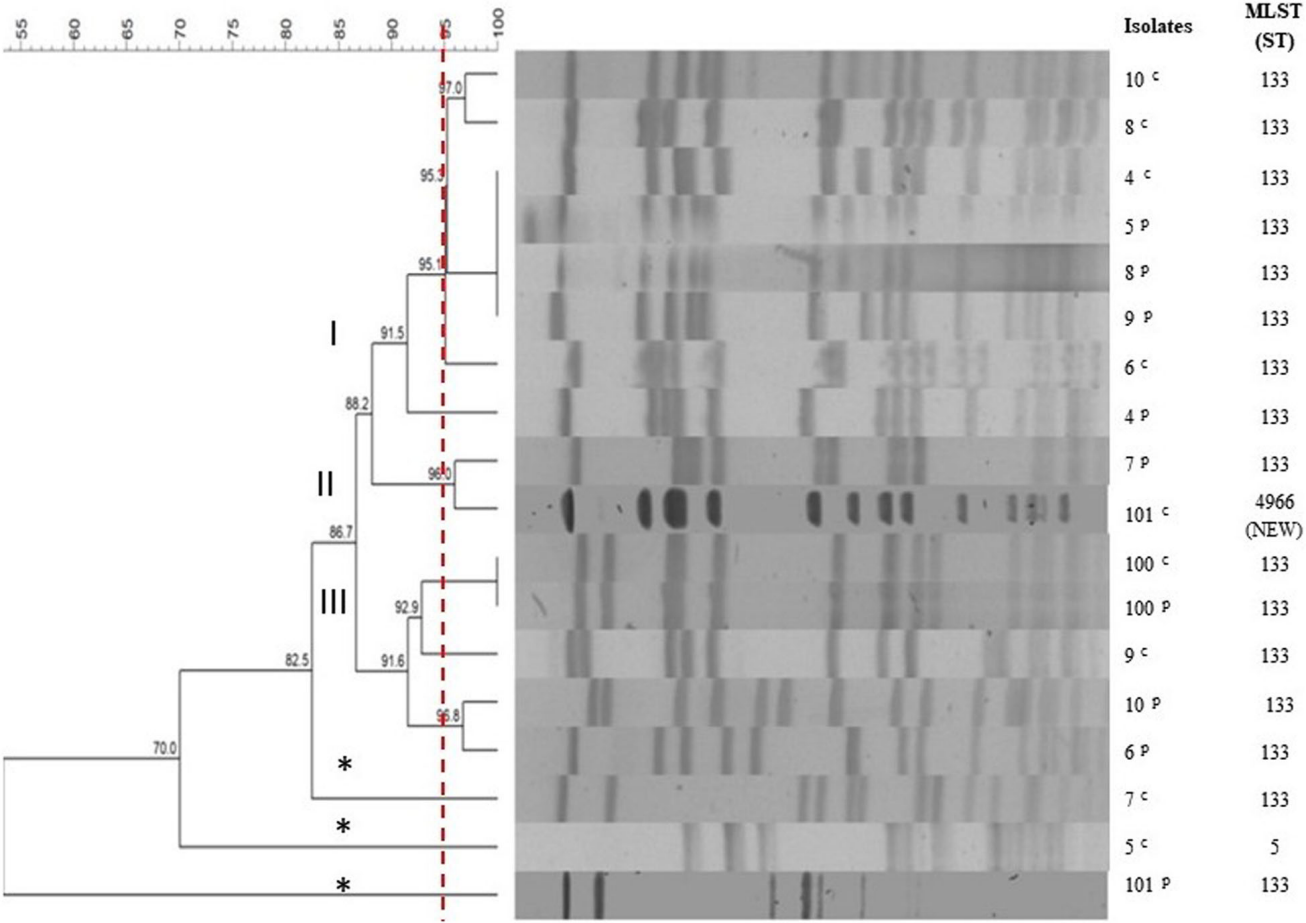
Dendrogram of Pulsed Field Gel Electrophoresis of 18 *Staphylococcus aureus* isolates and your Sequence Types. Dendogram generated by the UPGMA/Dice (Bionumerics, Applied Maths). *S. aureus* isolates from the animals 4, 8 and 100, before and after treatment, presented identical pulse types (95% similarity and 5% tolerance and optimization). Sixteen isolates were within the same ST 133, the exceptions were *S. aureus* 5 and 101 from before treatment, which presented in ST 5 and a new ST 4966, respectively. c: isolates of goats with mastitis before treatment; p: isolates of goats with mastitis after treatment. Red dotted line: clusters (I-III); * Isolated not clustered in clusters

*S. aureus* isolates from the animals 4, 8 and 100, before and after treatment, presented identical pulse types, whereas others obtained from other animals, before and after treatment, were classified into distinct or even unclassified pulse types using 95% similarity and 5% tolerance and optimization, as shown in Fig. 1*.*

From the MLST, all *S. aureus* isolates belonged to the same ST 133, except *S. aureus* 5 and 101 isolated before treatment with enrofloxacin. Isolate 5^c^ belonged to ST 5 and *S. aureus* 101^c^ was classified as a novel ST 4966 (Fig. 1).

## Discussion

In goats mastitis, the most important bacterial genus is *Staphylococcus* [10, 11] and the severity of the infections caused by *S. aureus* in different types of mastitis is associated with its virulence factors which allow it to adhere to surface, invade or avoid the immune system, and cause harmful toxic effects to the host [12, 13].

In general, considering the same animal as origin, the MICs of the tested antimicrobials increased for the isolates obtained after treatment and in some cases changed the profile for resistance (Table 1). There was an increase in the MIC for enrofloxacin in five isolates obtained after treatment, whereas in four isolates, the MIC was maintained (Table 2). The mechanism of action of fluoroquinolones is to inhibit DNA gyrase and thus inhibit bacterial cell division. For beta-lactams and fluoroquinolones, the inhibition of replication plays a central role in the development of the persistence phenomenon [5]. Persisters are not as dormant as spores but are slow or very slow growers [14]. In addition, gene exchange events can be increased up to 100-fold in persistent *S. aureus* isolates [15].

Of the 10 resistance genes tested, seven were detected in *S. aureus* isolated before and after treatment, but with a greater detection in isolates after treatment (*p* < 0.05). The *tetM* and *tetK* genes are associated with tetracycline resistance. The gene *tetM* is located in conjugative transposons, whereas the *tetK* gene is located in a plasmid, which is the transfer of genes to other species of bacteria [16, 17]. The *blaZ* gene encoding penicillin resistance may be located both chromosomally and in plasmids [17].

In the present study, the *mecA* gene was detected before and after treatment. Expression of this gene confers resistance to methicillin and most beta-lactams. The gene *mecA* is located on a mobile genetic element ‘staphylococcal cassette chromosome *mec* (SCC*mec*) [18]. Methicillin-resistant *S. aureus* (MRSA) is an important human and animal pathogen that is linked to several infections. Recently, the isolation of MRSA from dairy cows with mastitis [19–21], raw milk and dairy products has been reported worldwide [22, 23], as well as in goats with mastitis [24, 25].

The *ermA* and *ermB* genes were found in our study, these genes are linked to resistance to macrolides, lincosamide, and streptogramin once they belong to erythromycin ribosome methylase family of genes (*ermA, ermB*, and *ermC*). These genes are widely distributed in isolates of *Staphylococcus* spp. from humans and animals and are located mainly in plasmids [26]. The presence of these genes is concerning due to the possibility of the transfer of these genes to other bacteria, or even the contamination to other animals, including humans with multiresistant bacteria.

The *ant(4′)-Ia* gene codes for aminoglycoside modifying enzymes (AME), conferring resistance to aminoglycosides. It was found in isolates before and after treatment, but in isolates from animals 5 and 7, it appeared only after treatment.

The major facilitator superfamily (MFS) is one of the oldest and most diverse efflux system family in *S. aureus*, with more than 1000 members. Since its discovery, MFS transporters have become the target of studies because of the ability to confer resistance to multiple drugs [27]. The presence of MFS is clinically relevant, mainly in Gram-positive bacteria, where the most important genes for *S. aureus norA*, *norB*, *norC* and *lmrS* are located in genome [28]. The *norA* and *norC* genes confer resistance to fluorquinolones, whereas the *lmrS* gene confers resistance to linezolid, phenicol (chloramphenicol, florfenicol), trimethoprim, erythromycin, kanamycin and fusidic acid [28]. The *norA*, *norC* and *lmrS* genes were similarly detected in *S. aureus* isolated from mastitis before and after treatment, and may be an important factor related to treatment failures and the persistence of caprine mastitis. In this study, the treatment was performed with enrofloxacin, antimicrobial for which the etiological agent was sensitive, and that has good distribution in the udder. On the other hand, enrofloxacin was known to be widely used on the property studied for the treatment of pneumonia in young goats.

The *mgrA* gene acts as a global regulator, regulating some virulence factors such as capsule synthesis and the gene expression of multidrug efflux systems [28]. This gene acts as a negative regulator for the *tet38*, *norB* and *norC* genes contributing to quinolone resistance [29]. In this study, the regulatory gene *mgrA* was not detected, suggesting that the efflux systems *tet38* and *norC* were active. The Tet38 efflux system is encoded by chromosomes and confers resistance to tetracycline. In addition, the presence of this system is related to increased *S. aureus* invasion in epithelial cells, indicating that it may play new roles, in addition to resistance to antimicrobial drugs [30].

Of the 16 virulence genes tested, nine were detected (Table 2). Alpha hemolysis (*hla, hlb* and *hlγ*) permeabilizes cell membranes, destroys macrophages and lymphocytes and alters platelet morphology. ETA and ETB exfoliative toxins hydrolyze desmoglein 1, a cadherin responsible for the integrity of the adhesive structures, resulting in skin exfoliation, and lead to the destruction of the epidermal barrier facilitating the efficient progression of infection [31].

Mørk and colleagues [32] observed the presence of *S. aureus* toxin genes obtained from healthy goats (71%) and goats with mastitis (86%), showing that the presence of enterotoxin-encoding genes is common in *S. aureus*. On the other hand, the SEC toxin affects the bovine immune response, resulting in immunosuppression, which promotes the persistence of *S. aureus* in the bovine mammary gland and contributions to chronic intramammary infection [33]. The *sec* gene was found in this study in three isolates before and after treatment, suggesting that this may play an important role in the virulence and persistence of this pathogen in the mammary gland of goats.

*S. aureus* 4^c^/4^p^, 8^c^/8^p^, and 100^c^/100^p^ obtained from the respective animals 4, 8, and 100, before and after enrofloxacin treatment, were present in pulse types, I, I, and III, respectively, and were within the same ST 133 (Fig. 1). which was previously associated with the persistence of subclinical bovine mastitis in Brazilian herds [34]. Differences in pulsotypes can alter the form of manifestation of the disease, since a given pulsotype can confer greater or lesser severity of symptoms and also be more or less associated with persistence [35]. In our study, the three pulsotypes found may be associated with persistence, but for some animals, the pulsotypes of isolates obtained from the same animal before and after treatment were different. In addition, the same resistance genes and multidrug efflux systems were found, as well as small point variations in MIC values among *S. aureus* isolated from the same animal before and after treatment (Tables 1 and 2). Regarding the presence of virulence genes, it was verified that *S. aureus* 8^c^ and 8^p^ have the same genes and in *S. aureus* 8 and 100 there was only one gene addition in *S. aureus* isolated after treatment (Table 2); thus, indicating that they may be the same isolate, which could not be eliminated in treatment and consequently resulted in persistent mastitis.

Previous studies in other geographic distributions of isolates have associated ST or Clonal Complex (CC) 133 with small ruminants [36–39]. *S. aureus* isolated from animals is commonly designated for host-specific ST, such as ST 133 from sheep, goats and cattle distributed in different countries [40–43]. We characterized 18 isolates of *S. aureus*, 16 *S. aureus* as ST 133, one as ST 5 and one new, ST 4966, not previously described. CC 5 or ST 5 is a common clonal complex of *S. aureus* [44] isolated from bovine mastitis [45], including in Brazil [46], buffalo milk [47], humans and milk and dairy products samples [23, 48].

Aires-de-Souza [49] proposed that CC 133 isolates may have evolved and adapted to small ruminants, originating from humans due to adaptive diversification of the genome resulting from allelic variation, a loss of genes or the horizontal acquisition of mobile genetic elements. In the case of isolate 5^c^ there was a modification in the allele corresponding to the *yqil* gene related to the metabolism of Acetylcoenzyme A acetyltransferase (Fig. 1). Isolate 101 was modified from all alleles, so it was not possible to classify it, thus resulting in a new ST. Although the other isolates (six before and six after treatment) had different pulsetypes, their resistance and virulence profiles were generally not significantly altered.

MLST provides an excellent tool for investigating the population structure of *S. aureus* globally [50]. Indeed, MLST provide data that can be compared on a global scale and allow typing of important *S. aureus* clones such as ST 5 and ST 133 involved in human and animal infections, that are non-typable by the standard PFGE method (using *sma*I) due to DNA methylation [51, 52]. Besides, some authors claim that the discriminatory ability of MLST is low when compared with other techniques such as PFGE [53].

The use of antimicrobials, even for therapeutic purposes, may induce the emergence of mutations, which may be related to the persistence phenomenon, by altering some of the profiles of etiological agents. Bacterial persistence is a phenomenon that involves the emergence of subpopulations of clonal groups that enter a dormant state and return to multiplication after drug withdrawal [54]. This may have occurred in the present study, where differences in resistance, virulence and clonal profiles were observed in *S. aureus* isolated from the same animals before and after treatment, even after a short treatment period (7 days). Cirz et al. [55] verified that, in the presence of ciprofloxacin, a fluoroquinolone, after 120 min of exposure, a rapid diversification of the *S. aureus* population occurs, inducing the mobilization of the prophage, as well as significant alterations to the metabolism, in addition to inducing the SOS response, leading to adaptive mutagenesis. Schelli et al. [56] found metabolic changes in response to stress in *S. aureus* isolates incubated with quinolones for a short time (after 6 h).

Of the seven major genes of *S. aureus* multidrug efflux systems, five were detected in the present study in *S. aureus* isolates before and after treatment. Thus, the hypothesis of the persistence of isolates, which are associated with a replication arrest, biofilm production, activation of efflux pumps and stimulation of mutation events and horizontal gene transfer (HGT) [5], is reinforced.

Whereas microbial persistence describes a phenomenon in which microorganisms are considered susceptible to drugs when tested outside the host but are able to survive within the body despite the use of the appropriate antimicrobial [57], once again the persistence theory of microorganism resulting in disease persistence is emphasized.

## Conclusion

This study demonstrated that *S. aureus* with certain clonal characteristics, resistance profiles and virulence possess abilities that may contribute to its persistence in mastitis, leading to severe infections and subsequent chronicity. In addition, it can be concluded that even using conventional mastitis treatment, with isolation and selection of antibiotic-sensitive in antibiogram, respecting the appropriate doses and application intervals, occasionally some profiles of the etiologic agent may be changed, contributing to the development of persistent mastitis.

## Methods

This project was approved by the Committee of Ethics in the Use of Animals of the Universidade Federal de Viçosa, CEUA / UFV, with study number 42/2014.

### *Animals and Staphylococcus aureus isolates*

Nine animals, four Saanen and five Alpine breed (specialized breeds for milk production), aged 2–4 years, with a body weight of approximately 50 kg, identified by numbers 4, 5, 6, 7, 8, 9, 10, 100, and 101 were selected. These goats, belonging to the goat farming sector, Animal Science Department, of the Universidade Federal de Viçosa. This sector is located in Viçosa, Minas Gerais, 20°46′22.8″S 42°51′10.8″W, with a Cwa climate according to Köppen climate classification. The animals are kept under intensive farming in a free stall regime, with a high-level mechanical milking system and automatic cleaning of milk pipes.

The animals selected were examined and diagnosed with clinical mastitis caused by *S. aureus.* After antibiogram results, these animals were treated with enrofloxacin (Kinetomax® – Bayer), with a dose of 5 mg/kg every 24 h, administered intramuscularly for seven consecutive days. Twenty-one days after the completion of treatment, these animals continued to have clinical mastitis. New milk samples were collected and *S. aureus* was isolated again. Thus, 18 isolates of *S. aureus* were obtained (nine before treatment and nine after treatment). All the isolates were identified by phenotypic (morphostaining and biochemical) and genotypic (*femA* gene detection by PCR and sequencing) methods [58], and stored at −80 °C in microtubes containing Heart Brain Infusion (BHI) broth with 20% glycerol.

After the experiment, the animals remained in their place of origin and returned to receive the treatment recommended by the technicians responsible for the sanitary management of the place.

*S. aureus* isolates were identified according to the animal number to which they were isolated and the letters C and P subscribed to the numbers means before and after treatment with enrofloxacin, respectively.

### Resistance profile

#### Minimum inhibitory concentration (MIC)

The minimum inhibitory concentrations (MICs) were assessed using the E-test® method (bioMerieux). The bacterial inoculum was prepared in Müeller-Hilton (MH) broth and the turbidity was adjusted to McFarland scale 0.5 (~ 1.5 × 10^8^ CFU/mL). The inoculum was spread on a plate containing MH agar, and the E-test strips were dispensed on the surface of the agar. The plates were then incubated at 37 °C for 24 h. After the incubation period, the plates were read and interpreted following the manufacturer’s guidelines and published cut-off points [8, 9]. *S. aureus* ATCC® 29213 was used as control. Were tested penicillin, oxacillin, ampicillin, gentamicin, tetracycline, ciprofloxacin, vancomycin and enrofloxacin, antimicrobial agents of importance in the treatment of mastitis. The mean of three replicates was used.

#### Virulence and resistance genes detection

DNA extraction was performed using the Wizard® Genomic DNA Purification Kit (Promega®), following the protocol described for Gram-positive bacteria, modified by the addition of 100 μL lysostaphin (100 μg/mL, Sigma®) and incubation at 37 °C, in a water bath for 45 min at the lysis stage.

PCR was used for detection of resistance, multidrug efflux system and virulence genes (Table 3). The PCRs was performed using 12.5 μL of Green Master Mix 2X (Promega Corp.), 10 μM of each primer (forward and reverse), 2 μL (~ 100 ng/μL) of DNA and nuclease-free water for the final volume of 25 μL for reaction. The virulence genes *seg + sei* and *seh + sej* were detected using multiplex PCR [65].

**Table 3:** Primers used in the detection of *Staphylococcus aureus* resistance, multidrug efflux system and virulence genes

Category	Gene	Primer	Sequence	Product (bp)	Reference
**Resistance**	*mecA*	*mecA - f*	CCTAGTAAAGCTCCGGAA	314	[59]
		*mecA - r*	CTAGTCCATTCGGTCCA		
	*Aac(6′)/aph(2′)*	*Aac(6′)/aph(2′) - f*	GAAGTACGCAGAAGAGA	491	[59]
		*Aac(6′)/aph(2′) - r*	ACATGGCAAGCTCTAGGA		
	*aph(3′)-IIIa*	*aph(3′)-IIIa - f*	AAATACCGCTGCGTA	242	[59]
		*aph(3′)-IIIa - r*	CATACTCTTCCGAGCAA′		
	*ant(4′)-Ia*	*ant(4′)-Ia - f*	AATCGGTAGAAGCCCAA	135	[59]
		*ant(4′)-Ia - r*	GCACCTGCCATTGCTA		
	*tet(M)*	*tet(M) - f*	AGTGGAGCGATTACAGAA	360	[60]
		*tet(M) - r*	CATATGTCCTGGCGTGCTTA		
	*tet(K)*	*tet(K) - f*	GTAGCGACAATAGGTAATAGT	158	[60]
		*tet(K) - r*	GTAGTGACAATAAACCTCCTA		
	*blaZ*	*blaZ - f*	ACTTCAACACCTGCTGCTTTC	173	[60]
		*blaZ - r*	TGACCACTTTTATCAGCAACC		
	*ermA*	*ermA - f*	TATCTTATCGTTGAGAAGGGATT	139	[61]
		*ermA - r*	CTACACTTGGCTTAGGATGAAA		
	*ermB*	*ermB - f*	CTATCTGATTGTTGAAGAAGGATT	142	[61]
		*ermB - r*	GTTTACTCTTGGTTTAGGATGAAA		
	*ermC*	*ermC - f*	CTTGTTGATCACGATAATTTCC	299	[61]
		*ermC - r*	ATCTTTTAGCAAACCCGTATTC		
**Multidrug Efflux Pump**	*tet38*	*tet38 - f*	TTCAGTTTGGTTATAGACAA	400	[61]
		*tet38 - r*	CGTAGAAATAAATCCACCTG		
	*norA*	*norA - f*	TGCAATTTCATATGATCAATCCC	150	[29]
		*norA - r*	AGATTGCAATTCATGCTAAATAT		
	*norB*	*norB - f*	ATAAGGTAAGATAACTAGCA	150	[29]
		*norB - r*	ATCTCTATTTGCCTCCCTATA		
	*norC*	*norC - f*	AAATGGTTCTAAGCGACCAA	200	[29]
		*norC - r*	ATAAATACCTGAAGCAACGC		
	*LmrS*	*LmrS - f*	TAAAGTTGAATTAACAAC	180	[30]
		*LmrS - r*	GCGGATCCTTAAAATTTC		
	*mgrA*	*mgrA - f*	CGAATTCATTCATGATTT	200	[61]
		*mgrA - r*	AAAGTTGATTGTTTATTAA		
	*msrA*	*msrA - f*	TCCAATCATAGCACAAAATC	163	[61]
		*msrA - r*	AATTCCCTCTATTTGGTGGT		
**Virulence**	*hla*	*hla - f*	CTGATTACTATCCAAGAAATTCGATTG	209	[62]
		*hla - r*	CTTTCCAGCCTACTTTTTTATCAGT		
	*fnbA*	*fnbA - f*	GTGAAGTTTTAGAAGGTGGAAAGAITAG	643	[63]
		*fnbA - r*	GCTCTTGTAAGACCATTTTTCTTCAC		
	*fnbB*	*fnbB - f*	GTAACAGCTAATGGTCGAATTGATACT	524	[63]
		*fnbB - r*	CAAGTTCGATAGGAGTACTATGTTC		
	*eta*	*eta - f*	ACTGTAGGAGCTAGTGCATTTGT	190	[64]
		*eta - r*	TGGATACTTTTGTCTATCTTTTTCATCAAC		
	*etb*	*etb - f*	CAGATAAAGAGCTTTATACACACATTAC	621	[64]
		*etb - r*	AGTGAACTTATCTTTCTATTGAAAAACACTC		
	*lukDE*	*lukDE - f*	TGAAAAAGGTTCAAAGTTGATACGAG	269	[64]
		*lukDE - r*	TGTATTCGATAGCAAAAGCAGTGCA		
	*tst*	*tst- f*	TTCACTATTTGTAAAAGTGTCAGACCCACT	180	[64]
		*tst - r*	TACTAATGAATTTTTTTATCGTAAGCCCTT		
	*sea*	*sea - f*	ACGATCAATTTTTACAG	544	[65]
		*sea - r*	TGCATGTTTTCAGAGTTAATC		
	*seb*	*seb - f*	GAATGATATTAATTCGCATC	416	[65]
		*seb - r*	TCTTTGTCGTAAGATAAACTTC		
	*sec*	*sec - f*	GACATAAAAGCTAGGAATTT	257	[65]
		*sec - r*	AAATCGGATTAACATTATCCA		
	*sed*	*sed - f*	CTAGTTTGGTAATATCTCCT	317	[65]
		*sed - r*	TAATGCTATATCTTATAGGG		
	*see*	*see - f*	TAGATAAAGTTAAAACAAG	170	[65]
		*see - r*	TAACTTACCGTGGACCCTTC		
	*seg*	*seg - f*	GTTAGAGGAGGTTTTATG	198	[65]
		*seg - r*	TTCCTTCAACAGGTGGAGA		
	*seh*	*seh - f*	CAACTGCTGATTTAGCTCAG	173	[65]
		*seh - r*	CCCAAACATTAGCACCA		
	*sei*	*sei - f*	GGCCACTTTATCAGGACA	328	[65]
		*sei - r*	AACTTACAGGCAGTCCA		
	*sej*	*sej - f*	GTTCTGGTGGTAAACCA	131	[65]
		*sej - r*	GCGGAACAACAGTTCTGA		

The reference strains of *S. aureus* FRI 100 (*sea*); ATCC 14458 (*seb*); ATCC 19095 (*sec, sec, seh* and *sei*), FRI 472 (*sed*) and FRI 326 (*see*) were used as positive controls and were provided by Fundação Osvaldo Cruz (Fiocruz-RJ, Brazil).

### Clonal profile

#### Pulsed field gel electrophoresis (PFGE)

Macro-restriction analyses of *S. aureus* DNA were performed following the protocol described by Spanamberg et al. [66], with some modifications. For the preparation of the plugs of the 18 isolates of *S. aureus* (nine before and nine after treatment), the isolates were inoculated in tryptone soy broth (TSB) and incubated at 37 °C for 16 h, until obtaining an optical density (OD) of 1 (ʎ = 590 nm). After an adjustment for OD, 150 μL of the bacterial suspension was transferred to micro tubes and centrifuged at 16,000×*g* for 5 min. The supernatant was discarded and the pellet was resuspended in 150 μL Cell Suspension Buffer + 7 μL lysostaphin (1 mg/mL) + 7 μL lysozyme (10 mg/mL) + 150 μL low melting agarose 1% and maintained at 50 °C. For enzymatic digestion, about 1/5 of the original plug was sectioned and added to properly identified 0.5 mL micro tubes. The plugs were subjected to an initial stabilization in 200 μL of 1X enzyme buffer (TE) for 10 min. After the removal of the buffer, 150 μL of 1X TE was added again, accompanied by 20 U restriction enzyme *Sma*I (Promega Corporation, Madison, USA), followed by incubation at 25 °C for 4 h.

The DNA present in the plugs was separated using a CHEF-DRIII apparatus (Bio-RadLaboratories, Hercules, CA, USA), according to the following run protocol: 40–100 s for 2 h, followed by 2–35 s for 20 h at an angle of 120°, 6 V/cm, in 0.5X TBE buffer maintained at 14 °C. Pulse Marker™ 50–1.000 kb (Sigma – Aldrich Co.) was used as a marker. The obtained gels were developed in an immersion bath with UniSafeDye® intercalating dye (Uniscience, Brazil), visualized in a transilluminator under ultraviolet light and photographed for further analysis. The obtained bands were analysed using BioNumerics v.6.6.4 software (AppliedMaths, Kortrijk, Belgium). For the analysis and interpretation of the results, a dendrogram was constructed using the unweighted pair group method with arithmetic mean (UPGMA) method, with a similarity coefficient of 95%, and a tolerance and optimization of 5% each [67].

#### Multi locus sequence typing (MLST)

Seven housekeeping genes were used: *arcC* (Carbamate kinase), *aroE* (shikimate dehydrogenase), *glpF* (glycerol kinase), *gmk* (guanylate kinase), *pta* (phosphate acetyltransferase), *tpi* (Triose phosphate isomerase) and *yqiL* (Acetyl coenzyme A) acetyl transferase) [68].

The MLST analysis was performed through PCR reactions, with each reaction containing 25 μL of Green Master Mix 2X GoTaq® (Promega Corp.), 10 pmol of each primer and 2 μL (100 ng/μL) DNA, completing the volume with free nuclease water to obtain a final volume of 50 μL. PCR was performed for an initial denaturation of 5 min at 95 °C, followed by 30 cycles of 55 °C for 1 min, extension at 72 °C for 1 min, and denaturation at 95 °C for 1 min, followed by one step of final extension from 72 °C for 5 min. We followed the protocol described by Enright et al. [68].

The amplified products were sent for sequencing at Macrogen Incorporation (Seoul, South Korea). The sequencing chromatograms were analysed and trimmed, selecting only the sequenced nucleotides with Phred scores greater > 20 (accuracy > 99). Then, contigs of the nucleotide sequences were assembled using Geneious Prime version 2019.0. Subsequently, the sequences were aligned using Multiple Sequence Alignment - CLUSTALW with the software MEGA 7.0.21**.** Allele profiles, sequence types (STs) and clonal complexes were assigned using the MLST database (https://pubmlst.org/saureus/). Alleles and STs that had not been previously described were submitted to the database and were assigned as a new allele numbers and STs.

### Statistical analyses

The presence/absence ratio of the virulence genes, resistance and multidrug efflux system (explanatory variables) with mastitis before and after treatment (response variable) were analysed by descriptive statistics and Multinomial logistic regression. Initially, a univariate logistic regression analysis was performed; the genes that had a significant effect (*p* < 0.05) were analysed by multivariate logistic regression, and only the genes with a significant effect (p < 0.05) were retained in the final model. The explanatory variables that did not present convergence problems in the logistic regression were evaluated by the Fisher-Freeman-Halton test. For the MIC data, descriptive statistics were used, based on the mean of three replicates. All analyses were performed using SAS version 9.3 (SAS Institute Inc., Cary, NC).

## Data Availability

The datasets used and/or analysed during the current study are available from the corresponding author on reasonable request.

## Abbreviations

AMP: Ampicillin
CC: Clonal Complex
CFU: Colony-forming unit
CIP: Ciprofloxacin
CLSI: Clinical and Laboratory Standards Institute
ENR: Enrofloxacin
GEN: Gentamicin
MFS: Major facilitator superfamily
MH: Müeller–Hinton
MIC: Minimum inhibitory concentration
MLST: Multilocus sequence typing
OD: Optical density
OXA: Oxacillin
PCR: Polymerase Chain Reaction
PEN: Penicillin
PFGE: Pulsed-field gel electrophoresis
TET: Tetracycline
VAN: Vancomycin
